# Supplementary material for: Neurovascular coupling and bilateral connectivity during NREM and REM sleep
Source: eLife. 2020 Oct 29;9:e62071. doi: 10.7554/eLife.62071 (PMC7758068; doi:10.7554/eLife.62071)
Supplement: Supplementary file 1. [file elife-62071-supp1.docx]

**Title:** Neurovascular coupling and bilateral connectivity during NREM and REM sleep

**Authors:** Kevin L. Turner, Kyle W. Gheres, Elizabeth A. Proctor, Patrick J. Drew

**Supplemental Table 1 | Duration of each arousal state from each animal used in IOS experiments.**

| **Animal ID** | **Total Data (Hours)** | **Awake Data (Hours)** | **Awake Data (% of Total)** | **NREM Data (Hours)** | **NREM Data (% of Total)** | **REM Data (Hours)** | **REM Data**  **(% of Total)** |
| --- | --- | --- | --- | --- | --- | --- | --- |
| ***T99*** | 19.8 | 6.4 | 32.2 | 11.4 | 57.7 | 2.0 | 10.1 |
| ***T101*** | 18.8 | 12.6 | 66.8 | 5.2 | 27.6 | 1.1 | 5.6 |
| ***T102*** | 20.3 | 12.9 | 63.4 | 6.3 | 31.0 | 1.1 | 5.6 |
| ***T103*** | 21.0 | 15.2 | 72.4 | 4.8 | 22.9 | 1.0 | 4.7 |
| ***T105*** | 20.5 | 12.4 | 60.5 | 7.8 | 38.0 | 0.3 | 1.4 |
| ***T108*** | 18.8 | 12.2 | 65.0 | 6.0 | 32.1 | 0.5 | 2.9 |
| ***T109*** | 19.8 | 16.0 | 81.0 | 3.4 | 17.1 | 0.4 | 1.9 |
| ***T110*** | 16.0 | 13.0 | 81.5 | 2.6 | 16.4 | 0.3 | 2.1 |
| ***T111*** | 21.3 | 12.2 | 57.5 | 7.5 | 35.3 | 1.5 | 7.2 |
| ***T119*** | 29.5 | 19.4 | 65.8 | 7.1 | 24.1 | 3.0 | 10.1 |
| ***T120*** | 28.8 | 13.8 | 47.9 | 13.6 | 47.1 | 1.4 | 5.0 |
| ***T121*** | 27.5 | 10.2 | 37.2 | 15.2 | 55.4 | 2.0 | 7.4 |
| ***T122*** | 27.3 | 7.0 | 25.7 | 18.4 | 67.5 | 1.9 | 6.8 |
| ***T123*** | 32.8 | 10.9 | 33.2 | 21.5 | 65.5 | 0.4 | 1.3 |

**Supplemental Table 2 | Duration of each arousal state from each animal used in two photon experiments.**

| **Animal ID +**  **Arteriole ID** | **Total Data (Minutes)** | **Baseline**  **Diameter (µm)** | **Awake Data (Minutes)** | **NREM Data (Minutes)** | **REM Data (Minutes)** |
| --- | --- | --- | --- | --- | --- |
| ***T115 A1*** | 105.0 | 30.6 | 88.9 | 13.3 | 2.8 |
| ***T115 A2*** | 120.0 | 24.5 | 105.7 | 14.3 | 0.0 |
| ***T115 A3*** | 120.1 | 12.7 | 109.3 | 10.8 | 0.0 |
| ***T115 A4*** | 30.0 | 23.5 | 28.4 | 1.6 | 0.0 |
| ***T115 P1*** | 90.0 | 16.3 | 88.2 | 1.8 | 0.0 |
| ***T116 A1*** | 90.1 | 24.9 | 55.3 | 34.8 | 0.0 |
| ***T116 A2*** | 150.0 | 22.8 | 106.7 | 43.3 | 0.0 |
| ***T116 A3*** | 105.0 | 20.8 | 88.8 | 16.2 | 0.0 |
| ***T117 A1*** | 90.1 | 26.3 | 70.8 | 18.1 | 1.2 |
| ***T117 A2*** | 60.0 | 18.5 | 52.3 | 7.7 | 0.0 |
| ***T117 A3*** | 75.0 | 15.9 | 55.2 | 19.8 | 0.0 |
| ***T117 A4*** | 15.0 | 17.7 | 14.2 | 0.8 | 0.0 |
| ***T118 A1*** | 30.0 | 22.6 | 13.6 | 13.8 | 2.6 |
| ***T118 A2*** | 60.0 | 19.0 | 42.2 | 13.8 | 4.0 |
| ***T118 A3*** | 90.0 | 20.6 | 58.1 | 27.6 | 4.3 |
| ***T118 P1*** | 15.0 | 27.3 | 13.4 | 1.6 | 0.0 |
| ***T125 A1*** | 60.0 | 22.5 | 54.1 | 5.9 | 0.0 |
| ***T125 A2*** | 75.1 | 19.3 | 60.8 | 14.3 | 0.0 |
| ***T125 A3*** | 45.0 | 17.5 | 43.2 | 1.8 | 0.0 |
| ***T125 A4*** | 75.0 | 17.7 | 58.0 | 15.0 | 2.0 |
| ***T125 A5*** | 75.0 | 17.9 | 63.3 | 10.1 | 1.6 |
| ***T125 P1*** | 30.1 | 14.6 | 18.2 | 9.3 | 2.6 |
| ***T126 A1*** | 75.1 | 31.5 | 72.8 | 2.3 | 0.0 |
| ***T126 A2*** | 75.0 | 23.7 | 73.6 | 1.4 | 0.0 |
| ***T126 A3*** | 90.0 | 25.2 | 88.2 | 1.8 | 0.0 |
| ***T126 A4*** | 105.0 | 21.8 | 93.1 | 10.6 | 1.3 |
| ***T126 A5*** | 60.0 | 18.6 | 46.3 | 12.1 | 1.6 |
| ***T126 A6*** | 45.0 | 18.3 | 44.3 | 0.7 | 0.0 |
| ***T126 P1*** | 45.0 | 12.0 | 42.4 | 2.6 | 0.0 |

**Supplemental Table 3 | IOS arousal state classification criteria**

| **Arousal state** | **Color** | **Duration** | **Origin** | **Criterion** |
| --- | --- | --- | --- | --- |
| rfc-Awake | Light black | 5 seconds | Random forest classifier | rfc-Awake periods were denoted by moderate cortical gamma band power, low cortical delta band power, moderate-high whisker motion, moderate-high heart rate, and high EMG power. |
| rfc-NREM | Cyan | 5 seconds | Random forest classifier | rfc-NREM periods were denoted by moderate cortical gamma band power, high cortical delta band power, little-no whisker motion, low heart rate, and low EMG power. |
| rfc-REM | Dark red | 5 seconds | Random forest classifier | rfc-REM periods were denoted by high cortical gamma band power, high hippocampal theta band power, moderate whisker motion, moderate heart rate, and very low EMG power. |
| Awake Rest | Green | ≥ 10 seconds | Subsets of  rfc-Awake | Large epochs (typically > 60 seconds) were manually verified to be truly awake. Awake Rest was defined as no body movement or whisker motion and occurred at least 5 seconds away from a whisker stimulation. |
| Awake Whisking | Dark blue | 0-5 seconds post whisk | Subsets of  rfc-Awake | Large epochs (typically > 60 seconds) were manually verified to be truly awake. Awake Whisking was defined as whisking motion that lasted between 2 and 5 seconds long and occurred at least 5 seconds away from a whisker stimulation. |
| Awake Stimulation | Pink | 1-2 seconds post stim | Subsets of  rfc-Awake | Large epochs (typically > 60 seconds) were manually verified to be true awake behavior. Awake Stimulation was defined as a directed air puff (0.1 seconds, 10 PSI) to a contralateral whisker pad. |
| Contiguous NREM | Purple | ≥ 30 seconds | Subsets of  rfc-NREM | rfc-NREM periods that meet duration threshold and occurred in the absence of whisker stimulation |
| Contiguous REM | Orange | ≥ 60 seconds | Subsets of  rfc-REM | rfc-REM periods that meet duration threshold and occurred in the absence of whisker stimulation. Long REM events that were broken up by ≤ 10 seconds of misclassification were linked as a single event. |
| Alert | Gold | 15 minutes | Random forest classifier | Full 15-minute recordings with ≥ 80% rfc-Awake classifications and lacked whisker stimulation. |
| Asleep | Light blue | 15 minutes | Random forest classifier | Full 15-minute recordings with ≥ 80% rfc-NREM or rfc-REM classifications and lacked whisker stimulation. i.e. ≤ 20% rfc-Awake classification. |
| All Data | Brown | 15 minutes | Random forest classifier | All 15-minute recordings that lacked whisker stimulation regardless of rfc classifications. |

**Supplemental Table 4 | 2-photon arousal state classification criteria**

| **Arousal state** | **Color** | **Duration** | **Origin** | **Criterion** |
| --- | --- | --- | --- | --- |
| m-Awake | Light black | 5 seconds | Manually classified | m-Awake periods were denoted by moderate cortical gamma band power, low cortical delta band power, moderate-high whisker motion, moderate-high heart rate, and high EMG power. |
| m-NREM | Cyan | 5 seconds | Manually classified | m-NREM periods were denoted by moderate cortical gamma band power, high cortical delta band power, little-no whisker motion, low heart rate, and low EMG power. |
| m-REM | Dark red | 5 seconds | Manually classified | m-REM periods were denoted by high cortical gamma band power, high hippocampal theta band power, moderate whisker motion, moderate heart rate, and very low EMG power. |
| Awake Rest | Green | ≥ 10 seconds | Subsets of  m-Awake | Epochs were manually verified to be truly awake. Awake Rest was defined as no body movement or whisker motion. |
| Awake Whisking | Dark blue | 0-5 seconds post whisk | Subsets of  m-Awake | Epochs were manually verified to be truly awake. Awake Whisking was defined as whisking motion that lasted between 2 and 5 seconds long. |
| Contiguous NREM | Purple | ≥ 30 seconds | Subsets of  m-NREM | m-NREM periods that meet duration threshold. |
| Contiguous REM | Orange | ≥ 60 seconds | Subsets of  m-REM | m-REM periods that meet duration threshold. |
| Alert | Gold | 15 minutes | Manually classified | Full 15-minute recordings with ≥ 80% m-Awake classifications. |
| All Data | Brown | 15 minutes | Manually classified | All 15-minute recordings regardless of classification (entire data set). |

**Supplemental Table 5 | Spectral power in delta band, theta band, alpha band, and beta band at 0.1 Hz**

| **Spectral Power at 0.1 Hz** | **Awake Rest** | **Cont. NREM** | **Cont. REM** | **Alert** | **Asleep** | **All Data** |
| --- | --- | --- | --- | --- | --- | --- |
| Delta band Power (a.u.) | 0.9 ± 0.1 | 4.7 ± 3.2  (p < 4.1×10^-10^) | 3.5 ± 1.8  (p < 1.2×10^-5^) | 1.1 ± 0.4  (p < 0.8) | 5 ± 3.3  (p < 2.9×10^-11^) | 3 ± 1.7  (p < 0.0003) |
| Theta band Power (a.u.) | 0.9 ± 0.1 | 19.2 ± 59.9  (p < 0.08) | 27.6 ± 72.6  (p < 0.01) | 1 ± 0.3  (p < 0.99) | 2.5 ± 1.7  (p < 0.88) | 1.7 ± 0.6  (p < 0.94) |
| Alpha band Power (a.u.) | 0.9 ± 0.1 | 59.8 ± 177  (p < 0.008) | 47.3 ± 100  (p < 0.04) | 1.2 ± 0.4  (p < 0.99) | 6.5 ± 4.2  (p < 0.8) | 3.9 ± 1.9  (p < 0.89) |
| Beta band Power (a.u.) | 0.9 ± 0.1 | 123.9 ± 397.6  (p < 0.01) | 69.6 ± 170.3  (p < 0.15) | 1.3 ± 0.6  (p < 0.99) | 7.7 ± 4.8  (p < 0.89) | 4.6 ± 2.5  (p < 0.94) |

Mean ± 1 standard deviation. p-values as a comparison to "Rest".

**Supplemental Table 6 | Spectral power in delta band, theta band, alpha band, and beta band at 0.01 Hz**

| **Spectral Power at 0.01 Hz** | **Alert** | **Asleep** | **All Data** |
| --- | --- | --- | --- |
| Delta band Power (a.u.) | 8.3 ± 6 | 21.3 ± 21.3  (p < 0.004) | 18.7 ± 16.3  (p < 0.02) |
| Theta band Power (a.u.) | 5.5 ± 2.7 | 20.9 ± 18.3  (p < 8.6×10^-6^) | 13.8 ± 7.5  (p < 0.01) |
| Alpha band Power (a.u.) | 6.7 ± 4.5 | 62.5 ± 40.7  (p < 3.9×10^-10^) | 41.2 ± 24.8  (p < 2.2×10^-5^) |
| Beta band Power (a.u.) | 10.8 ± 10 | 91.4 ± 60.9  (p < 6.9×10^-10^) | 62.7 ± 35.9  (p < 1.5×10^-5^) |

Mean ± 1 standard deviation. p-values as a comparison to "Alert".

**Supplemental Table 7 | Magnitude of Coherence^2^ of bilateral delta band, theta band, alpha band, and beta band at 0.1 Hz**

| **Coherence^2^ at 0.1 Hz** | **Awake Rest** | **Cont. NREM** | **Cont. REM** | **Alert** | **Asleep** | **All Data** |
| --- | --- | --- | --- | --- | --- | --- |
| Delta band (Coherence^2^) | 0.06 ± 0.06 | 0.07 ± 0.06  (p < 0.59) | 0.03 ± 0.03  (p < 0.03) | 0.11 ± 0.08  (p < 0.002) | 0.08 ± 0.06  (p < 0.37) | 0.07 ± 0.05  (p < 0.63) |
| Theta band (Coherence^2^) | 0.05 ± 0.04 | 0.08 ± 0.07  (p < 0.08) | 0.09 ± 0.06  (p < 0.03) | 0.14 ± 0.09  (p < 1.2×10^-5^) | 0.07 ± 0.06  (p < 0.18) | 0.08 ± 0.05  (p < 0.13) |
| Alpha band (Coherence^2^) | 0.02 ± 0.02 | 0.19 ± 0.06  (p < 8.7×10^-17^) | 0.1 ± 0.05  (p < 6.1×10^-6^) | 0.09 ± 0.04  (p < 8.2×10^-5^) | 0.07 ± 0.05  (p < 0.003) | 0.07 ± 0.03  (p < 0.008) |
| Beta band (Coherence^2^) | 0.08 ± 0.05 | 0.39 ± 0.1  (p < 1.4×10^-30^) | 0.21 ± 0.08  (p < 5×10^-11^) | 0.16 ± 0.08  (p < 1.9×10^-6^) | 0.16 ± 0.07  (p < 3.4×10^-6^) | 0.17 ± 0.06  (p < 3.9×10^-7^) |

Mean ± 1 standard deviation. p-values as a comparison to "Rest".

**Supplemental Table 8 | Magnitude of Coherence^2^ of bilateral delta band, theta band, alpha band, and beta band at 0.01 Hz**

| **Coherence^2^ at 0.01 Hz** | **Alert** | **Asleep** | **All Data** |
| --- | --- | --- | --- |
| Delta band (Coherence^2^) | 0.68 ± 0.16 | 0.34 ± 0.23  (p < 2.8×10^-11^) | 0.54 ± 0.22  (p < 5.5×10^-5^) |
| Theta band (Coherence^2^) | 0.6 ± 0.19 | 0.59 ± 0.26  (p < 0.67) | 0.59 ± 0.25  (p < 0.53) |
| Alpha band (Coherence^2^) | 0.62 ± 0.17 | 0.74 ± 0.13  (p < 0.0007) | 0.74 ± 0.13  (p < 0.001) |
| Beta band (Coherence^2^) | 0.74 ± 0.12 | 0.8 ± 0.11  (p < 0.08) | 0.81 ± 0.1  (p < 0.05) |

Mean ± 1 standard deviation. p-values as a comparison to "Alert".

**Supplemental Table 9 | Pearson’s correlation coefficients of bilateral delta band, theta band, alpha band, and beta band**

| **Pearson’s Correlation Coef.** | **Awake Rest** | **Whisking** | **Cont. NREM** | **Cont. REM** | **Alert** | **Asleep** | **All Data** |
| --- | --- | --- | --- | --- | --- | --- | --- |
| Delta band (R) | 0.15 ± 0.05 | 0.17 ± 0.08  (p < 0.28) | 0.17 ± 0.09  (p < 0.27) | 0.08 ± 0.05  (p < 0.0002) | 0.32 ± 0.08  (p < 1.4×10^-14^) | 0.19 ± 0.09  (p < 0.03) | 0.26 ± 0.1  (p < 2.1×10^-8^) |
| Theta band (R) | 0.14 ± 0.06 | 0.26 ± 0.09  (p < 1.2×10^-8^) | 0.15 ± 0.06  (p < 0.68) | 0.14 ± 0.07  (p < 0.89) | 0.3 ± 0.1  (p < 5.7×10^-13^) | 0.24 ± 0.1  (p < 5.7×10^-7^) | 0.28 ± 0.09  (p < 8.4×10^-11^) |
| Alpha band (R) | 0.09 ± 0.04 | 0.18 ± 0.08  (p < 1.9×10^-6^) | 0.2 ± 0.05  (p < 6.2×10^-9^) | 0.14 ± 0.05  (p < 0.002) | 0.25 ± 0.04  (p < 8.7×10^-15^) | 0.33 ± 0.04  (p < 3×10^-24^) | 0.31 ± 0.03  (p < 1.5×10^-23^) |
| Beta band (R) | 0.15 ± 0.06 | 0.14 ± 0.1  (p < 0.45) | 0.36 ± 0.06  (p < 3.6×10^-23^) | 0.25 ± 0.07  (p < 5.5×10^-9^) | 0.33 ± 0.08  (p < 5.7×10^-19^) | 0.46 ± 0.06  (p < 4.3×10^-33^) | 0.45 ± 0.06  (p < 3.3×10^-33^) |

Mean ± 1 standard deviation. p-values as a comparison to "Rest".

**Supplemental Table 10 | Coherence of ∆[HbT] vs. delta band, theta band, alpha band, and beta band at 0.1 Hz**

| **Coherence^2^ at 0.1 Hz** | **Awake Rest** | **Cont. NREM** | **Cont. REM** | **Alert** | **Asleep** | **All Data** |
| --- | --- | --- | --- | --- | --- | --- |
| ∆[HbT]-Delta (Coherence^2^) | 0.05 ± 0.04 | 0.07 ± 0.06  (p < 0.15) | 0.02 ± 0.02  (p < 0.003) | 0.06 ± 0.06  (p < 0.52) | 0.07 ± 0.06  (p < 0.17) | 0.06 ± 0.04  (p < 0.21) |
| ∆[HbT]-Theta (Coherence^2^) | 0.01 ± 0.01 | 0.11 ± 0.06  (p < 5.1×10^-20^) | 0.06 ± 0.03  (p < 6.2×10^-6^) | 0.02 ± 0.02  (p < 0.56) | 0.06 ± 0.04  (p < 1.3×10^-6^) | 0.03 ± 0.03  (p < 0.03) |
| ∆[HbT]-Alpha (Coherence^2^) | 0.02 ± 0.02 | 0.18 ± 0.05  (p < 1.5×10^-37^) | 0.04 ± 0.03  (p < 0.009) | 0.02 ± 0.02  (p < 0.8) | 0.07 ± 0.06  (p < 2.1×10^-6^) | 0.04 ± 0.02  (p < 0.03) |
| ∆[HbT]-Beta (Coherence^2^) | 0.04 ± 0.03 | 0.28 ± 0.06  (p < 4.3×10^-57^) | 0.05 ± 0.04  (p < 0.37) | 0.04 ± 0.04  (p < 0.82) | 0.11 ± 0.04  (p < 8.9×10^-11^) | 0.09 ± 0.03  (p < 1.2×10^-7^) |
| ∆[HbT]-Gamma (Coherence^2^) | 0.18 ± 0.11 | 0.32 ± 0.09  (p < 7.9×10^-10^) | 0.08 ± 0.07  (p < 1.8×10^-5^) | 0.27 ± 0.14  (p < 6×10^-5^) | 0.28 ± 0.14  (p < 7.6×10^-6^) | 0.28 ± 0.13  (p < 4.1×10^-6^) |

Mean ± 1 standard deviation. p-values as a comparison to "Rest".

**Supplemental Table 11 | Coherence of ∆[HbT] vs. delta band, theta band, alpha band, and beta band at 0.01 Hz**

| **Coherence at 0.01 Hz** | **Alert** | **Asleep** | **All Data** |
| --- | --- | --- | --- |
| ∆[HbT]-Delta (Coherence) | 0.1 ± 0.11 | 0.14 ± 0.12  (p < 0.05) | 0.2 ± 0.11  (p < 2×10^-5^) |
| ∆[HbT]-Theta (Coherence) | 0.11 ± 0.1 | 0.5 ± 0.24  (p < 7.9×10^-14^) | 0.42 ± 0.19  (p < 1.2×10^-10^) |
| ∆[HbT]-Alpha (Coherence) | 0.14 ± 0.13 | 0.39 ± 0.19  (p < 2×10^-11^) | 0.43 ± 0.14  (p < 4.2×10^-15^) |
| ∆[HbT]-Beta (Coherence) | 0.16 ± 0.18 | 0.52 ± 0.18  (p < 1.7×10^-16^) | 0.55 ± 0.13  (p < 7.9×10^-19^) |
| ∆[HbT]-Gamma (Coherence) | 0.38 ± 0.18 | 0.46 ± 0.16  (p < 0.06) | 0.51 ± 0.14  (p < 0.001) |

Mean ± 1 standard deviation. p-values as a comparison to "Alert".

**Supplemental Table 12 | Model OOB error vs. shuffled-date model OOB error for each IOS animal**

| **Animal ID** | **Random Forest Out-of-bag (OOB) error (%)** | **Mean OOB (%) of 100 shuffled data models** |
| --- | --- | --- |
| ***T99*** | 7.5 | 47.1 |
| ***T101*** | 7.2 | 32.8 |
| ***T102*** | 6.3 | 33.0 |
| ***T103*** | 5.6 | 25.8 |
| ***T105*** | 6.0 | 31.3 |
| ***T108*** | 8.1 | 45.3 |
| ***T109*** | 4.6 | 22.6 |
| ***T110*** | 5.2 | 20.7 |
| ***T111*** | 8.3 | 39.9 |
| ***T119*** | 7.5 | 39.8 |
| ***T120*** | 8.7 | 52.1 |
| ***T121*** | 9.3 | 53.4 |
| ***T122*** | 7.5 | 35.1 |
| ***T123*** | 7.2 | 26.2 |
